# Automated Assessment of Hypernasality in Motor Speech Disorders Using Nasal Cognate Distinctiveness and Nasal Posterior Probability

**DOI:** 10.64898/2026.09.19.26363478

**Published:** 2026-09-21

**Authors:** Fenqi Wang, Rene L. Utianski, Leland R. Barnard, Joseph R. Duffy, Hugo Botha

## Abstract

**Purpose:**

To evaluate whether two automated measures of nasality, Nasal Cognate Distinctiveness (NCD) and nasal posterior probability (NPP), provide clinically meaningful markers of hypernasality in speakers with motor speech disorders (MSDs) and to determine whether hypernasality-related information extends beyond stop consonants, which are typically targeted in cognate-based approaches.

**Method:**

Sentence-level speech recordings were obtained from 561 patients, including 374 neurologically typical controls and 187 individuals with MSDs. NCD was computed for voiceless stops (/p/, /t/, /k/) using likelihood ratios between stops and their nasal cognates. NPP was extracted using a phonological feature-based neural network model and analyzed for stops, consonants, and vowels. Hypernasality severity was rated by speech-language pathologists. Group differences and associations with hypernasality severity were evaluated using linear mixed-effects models and Spearman correlations, and receiver operating characteristic (ROC) analyses were used to evaluate discrimination of absent versus present hypernasality. An additional 20 controls with repeat recordings were used to evaluate test-retest reliability and minimal detectable change (MDC).

**Results:**

NCD generally decreased and NPP increased with increasing hypernasality severity, with phoneme-specific severity effects observed for NCD. Across stops, NCD and NPP were strongly inversely correlated and showed comparable discrimination of absent versus present hypernasality (AUCs = 0.796 and 0.730, respectively). Consonant and vowel NPP measures were both positively associated with hypernasality severity and demonstrated identical classification performance (AUC = 0.853). No significant differences in ROC performance were observed between NCD and NPP or between consonant and vowel NPP measures. Test-retest change exceeded the MDC threshold in 2 of 20 controls for NCD and 1 of 20 for NPP.

**Conclusions:**

Both NCD and NPP provide objective markers of hypernasality in speakers with MSDs. The measures capture related aspects of nasalization and demonstrate comparable ability to identify clinically meaningful hypernasality. NPP captures hypernasality-related acoustic information across both consonants and vowels, with similar relationships to severity and classification performance across sound classes. Test-retest findings provide preliminary MDC benchmarks for interpreting longitudinal change.

## Full Text Availability

The license terms selected by the author(s) for this preprint version do not permit archiving in PMC. The full text is available from the preprint server.

